# Possible Effects of Acupuncture in Poststroke Aphasia

**DOI:** 10.1155/2023/9445381

**Published:** 2023-04-12

**Authors:** Bifang Zhuo, Shizhe Deng, Boxuan Li, Weiming Zhu, Menglong Zhang, Chenyang Qin, Zhihong Meng

**Affiliations:** ^1^First Teaching Hospital of Tianjin University of Traditional Chinese Medicine, Tianjin, China; ^2^National Clinical Research Center for Chinese Medicine Acupuncture and Moxibustion, Tianjin, China

## Abstract

Neural plasticity promotes the reorganization of language networks and is an essential recovery mechanism for poststroke aphasia (PSA). Neuroplasticity may be a pivotal bridge to elucidate the potential recovery mechanisms of acupuncture for aphasia. Therefore, understanding the neuroplasticity mechanism of acupuncture in PSA is crucial. However, the underlying therapeutic mechanism of neuroplasticity in PSA after acupuncture needs to be explored. Excitotoxicity after brain injury affects the activity of neurotransmitters and disrupts the transmission of normal neuron information. Thus, a helpful strategy of acupuncture might be to improve PSA by affecting the availability of these neurotransmitters and glutamate receptors at synapses. In addition, the regulation of neuroplasticity by acupuncture may also be related to the regulation of astrocytes. Considering the guiding significance of acupuncture for clinical treatment, it is necessary to carry out further study about the influence of acupuncture on the recovery of aphasia after stroke. This study summarizes the current research on the neural mechanism of acupuncture in treating PSA. It seeks to elucidate the potential effect of acupuncture on the recovery of PSA from the perspective of synaptic plasticity and integrity of gray and white matter.

## 1. Introduction

Poststroke aphasia (PSA), a language-acquired disorder, is one of the most devastating symptoms after stroke. PSA affects approximately one-third of stroke patients, and 30–43% of them suffer from long-term effects of aphasia [1, 2]. People with PSA have minimal community activities and often experience mental health problems, thus increasing the prevalence of depression and anxiety disorders in PSA patients [3]. In addition, patients with PSA often experience daytime sleepiness, fatigue, and decreased attention [4, 5]. Hence, PSA has a significant negative impact on quality of life. The symptoms after ischemic stroke are mainly caused by the death of neurons in the ischemic core, resulting in the destruction of normal neural circuits. Thus, promoting the recovery of PSA often involves the establishment of new neural circuits. Therefore, functional recovery in aphasia patients is related to neuroplasticity [6]. Neuroplasticity plays a vital role in adaptation, reorganization, self-repair, and learning and memory [7]. The recovery of neuroplasticity for aphasia after stroke can be summarized in the following two points [8, 9]: first one is the recovery and reorganization of the residual neural networks in the damaged brain area. Second one is the compensation of the neural pathways around focal tissue or the mirror areas of language function in the contralateral cerebral hemisphere (healthy cerebral hemisphere). Thus, the essence of language function recovery results from network reorganization, which is a complex process. However, systematic studies of the neural pathways and recovery mechanisms for aphasia remain lacking.

Acupuncture promotes the process of repairation of the nervous system by regulating neuroplasticity. As one of the ordinary means for PSA treatment, acupuncture has unique advantages in alleviating PSA. Acupuncture has the characteristics of evident efficacy, low price, flexible use, and few adverse reactions. According to traditional Chinese medicine theory, acupuncture applied to specific acupoints can dredge meridians, reconcile Yin and Yang, strengthen the body resistance, and dispel blocked energy by acupuncture on specific acupoints. Thus, acupuncture is one of the alternative methods that has been a favorable choice for PSA patients who need long-term treatment [10].

In this study, we summarize the effects of acupuncture that promoting the recovery of PSA patients and review the language processing of PSA and its regulation at the cellular and molecular levels to provide a complementary perspective, and promote neuroplasticity.

## 2. Poststroke Aphasia

### 2.1. Overview of PSA

PSA is mainly related to infarction in language function-related areas, especially in the middle cerebral artery (MCA). Lesions, such as hypertension and ischemia, cause arterial stenosis or occlusion in these related areas, reducing blood perfusion and blood flow in the penumbral area, and thus affecting the corresponding language function [11–14]. The significant damage of vascular occlusion leads to neural tissue infarction [15]. During cerebral ischemia, endothelin type B receptors are expressed in the vascular smooth muscle of the MCA, leading to vasoconstriction [16]. The endothelin-1 (ET-1) level in plasma and cerebrospinal fluid increases. The increase in local ET-1 concentration contracts the blood vessels in the ischemic areas and surrounding tissues, thus aggravating the degree of ischemia and the scope of tissue damage [17].

After a stroke, local ischemia and hypoxia lead to reduced production of adenosine triphosphate (ATP). Insufficient energy supports brain cells, resulting in the imbalance of the sodium–potassium pump (Na/K pump) and the plasma membrane calcium pump (Ca^2+^-ATP pump) on the cell membrane. Then, excessive neuronal depolarization, excitatory neurotransmitter release, and reduced neurotransmitter reuptake occur in the extracellular space, resulting in an increased intracellular calcium ion (Ca^2+^) concentration. Accumulation of calcium ions causes a downstream neurotoxic cascade, including calcium-dependent enzymes and hyperactivation of signaling, causing impaired neuronal function and irreversible damage [18–20]. This process leads to the blockage of neuronal information transmission and additional cell death, and transneuronal degeneration. In addition, focal infarction results in an altered state of activity in intact but remote brain regions due to abnormal connections that affect behavior [21].

Neuroinflammation is also involved in remote responses to focal cerebral ischemia and may further induce secondary brain injury [22, 23]. This biphasic induction of the inflammatory response peaks in both acute and subacute ischemic stroke [24]. Moreover, the mechanisms of brain tissue edema, metabolic disorders, and the impairment of tissue function are all associated with breaking aphasia-related language networks [25].

### 2.2. Structures Affected by PSA

Aphasia is usually associated with impaired left language networks, including the inferior frontal gyrus, angular gyrus, middle frontal gyrus, superior temporal gyrus, supramarginal gyrus, inferior temporal gyrus, middle temporal gyrus (MTG), and supplementary motor area [26]. The classical theories of aphasia first originated from the Wernicke–Geschwind model [27, 28]. Then, it is organized by the Geschwind [29] partition. The model mainly consists of the Wernicke area, Broca area, and the arcuate fasciculus (AF) connecting the two [30]. The Broca area primarily refers to the Broadman areas 44 and 45 (BA44 and BA45), namely, the posterior half of the left inferior frontal gyrus [31]. The Wernicke district refers mainly to the Broadman areas 22, 41, and 42 or the anterior part of the superior temporal gyrus [32, 33]. Of those, the Broca area is the sports center of language, so patients with impaired Broca area have impaired language fluency and cannot organize the correct language [34, 35]. The Wernicke area is the domain to understand written and spoken language, so patients with impairments in this area will have difficulty in comprehension or lose the ability for oral or written expression [36]. In addition, the AF, a neural channel connecting the Broca area and Wernicke area, has some connection with repeatability [37, 38].

However, with the development of modern neuroimaging techniques, the Wernicke–Geschwind model is no longer suitable for studying modern aphasia. In addition to these two regions, other neural pathways are involved in forming language functions [39]. The dual-stream language model proposed by Hickok and Poeppel suggests two language processing systems, ventral stream (semantic processing system) and dorsal stream (speech processing system), and is the primary model of aphasia [40]. The ventral stream involves bilateral tissues, despite computational differences between the left and right hemispheres [41], which mainly project ventrolaterally toward the posterior MTG and process speech signals to support auditory understanding, which consists of the inferior fronto-occipital fascicle (IFOF), uncinate fascicle, and inferior longitudinal tract [42]. IFOF is considered the central area of the ventral stream or direct-ventral pathway, connecting the posterior temporal and occipital lobes to the inferior frontal cortex and dorsolateral prefrontal cortex and facilitating auditory understanding [43]. Moreover, the dorsal stream is dominated by the left hemisphere, involves the parietal operculum, the posterior dorsal temporal lobe, and the posterior frontal lobe, and is mainly composed of the superior temporal gyrus/sulcus, posterior planum temporale (area Spt), the pars opercularis of Broca area, and dorsal premotor cortex, and eventually projects to the frontal area [44]. Its main function is language perception and language sensorimotor mapping. Area Spt, the posterior Sylvian fissure at the temporoparietal boundary, is a central region among these areas [45, 46]. Both speech perception and production systems converge in this region [47]. The concept of connectivity patterns and specific functional roles of these white matter (WM) fibers in PSA patients' language networks helps assess patient language dysfunction characteristics, severity, and outcomes, and guides clinical precision rehabilitation. In short, as a relay system, the language dual-stream model processes a higher-order language function. Its essence is to integrate language structure and function between cortical areas. Each area has a specific function [48], so different brain regions and their connection to neural pathway damage will exhibit different types of aphasia symptoms.

### 2.3. Receptors

After a stroke, brain cells, receptors, and neurotransmitters react accordingly and affect the process of nerve damage or neuroprotection.

**Figure 1:**
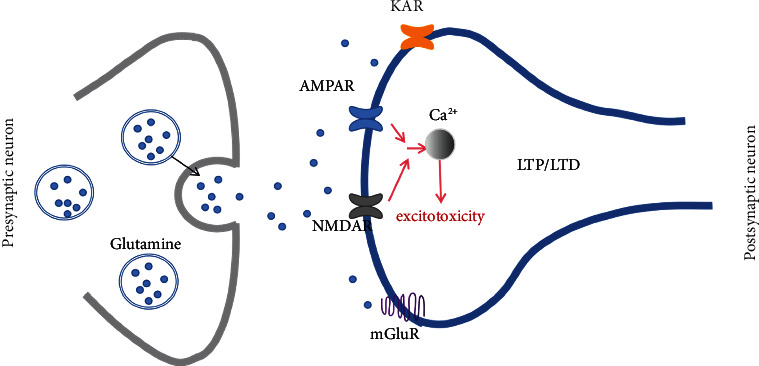
The transmission of glutamate. Glutamate mainly acts on glutamate receptors [AMPAR, NMDAR, kainic acid receptor (KAR), and metabolotropic glutamate receptors (mGluR)]. Commonly, glutamate receptors are involved in synaptic plasticity [long-term potentiation (LTP)/long-term depression (LTD)] and the transmission of excitatory neurotransmitters. After stroke, ischemia and hypoxia of the brain will lead to excessive activation of glutamate receptors, causing Ca^2+^ influx, and increased intracellular Ca^2+^ concentrations cause excitotoxicity and cytotoxic effects.

Although few studies directly illustrate the intrinsic link between glutamate and language function, existing studies have begun to elucidate the role of glutamate neuron conduction in language. As the primary neurotoxic excitatory neurotransmitter in the central nervous system (CNS), glutamate is the primary mediator of brain injury after stroke. After ischemic brain injury, glutamate activates receptors of *α*-amino-3-hydroxy-5-methyl-4-isoxazole-propionic acid (AMPA) and *N*-methyl-D-aspartate (NMDA), allowing Ca^2+^ to enter neurons, causing excitotoxicity [49, 50]. Therefore, the dysregulation of glutamate transmission causing disorders in the conduction and processing of subcortical information may be one of the neural mechanisms of language disorders after stroke. Disruption of the subcortical language network by excitotoxicity leads to impaired protrusion integrity and an inability to process relevant language information, especially at language processing centers with a high density of AMPA and NMDA receptors (AMPARs and NMDARs), thus triggering corresponding language barriers [51] (Figure 1).

AMPARs are involved in regulating learning and memory activities. The damage to gray matter (GM) and WM after excessive activation of AMPARs mainly cause damage to the glia, myelin, axons, and perinuclear bodies of neurons [52]. These injuries break the dysfunction of language information in the balance of neuronal transmission, thus affecting the function of the corresponding subcortical regions. AMPAR comprises GluA1, GluA2, GluA3, and GluA4 subunits. Because of the presence of GluA2, AMPAR does not permeate Ca^2+^. However, in the case of ischemia and hypoxia, the lack of GluA2 causes AMPARs to penetrate Ca^2+^ (CP-AMPARs) and causes excitotoxicity.

NMDAR-mediated excitotoxicity is an essential mechanism for causing brain injury. In most cases, the NMDA receptor is a heterotetramer composed of two GluN1 subunits and two GluN2 subunits. Three subunits constitute the NMDAR: NR 1, NR 2, and NR 3. NR 1 has eight different subunits, NR 2 has four different subunits, and NR 3 has two different subunits. NMDAR is distributed in the synaptic membrane and inside and outside the cell. NMDAR is linked to the macromolecular signaling complex by postsynaptic density protein 95, and is involved in postsynaptic membrane plasticity [53]. As with AMPARs, NMDARs have important implications for learning, memory, and brain development, even excessive activation leads to excitotoxicity. NMDAR has a dual regulatory effect on neuronal survival and apoptosis because of the different NMDAR subpopulations [54]. Activation of synaptic NMDARs (syn-NMDARs) promotes LTP, which favors neuronal survival while interfering with the expression of neuronal death mechanisms. Extrasynaptic NMDARs (eNMDARs) are the opposite of prominent NMDARs, activating LTD and death-signaling pathways in neurons [55].

Syn-NMDAR, mainly the GluN2A subunit, activates a downstream cascade reaction through Ca^2+^ influx to activate the neuronal survival signaling complex. Excessive activation of syn-NMDAR leads to excessive Ca^2+^ influx and triggers a series of downstream cascade reactions to activate eNMDAR [56]. Thus, hyperactivated pathological eNMDAR activates the neuronal death signaling complex and turns off the cyclic adenosine monophosphate (cAMP) response element-binding protein (CREB) signaling pathway, mediating AMPAR endocytosis and neuronal death to prevent abnormal neurons from excessive firing in the entire CNS. Therefore, syn-NMDAR itself does not cause cell death [57]. Furthermore, studies have shown that eNMDAR activation alone does not lead to neuronal death. The process of neuronal death is activated when eNMDAR is coactivated with synaptic NMDAR at sites on neurons. Moreover, the eNMDAR activation threshold is higher than the syn-NMDAR activation threshold. Thus, eNMDAR is the leading cause of neuronal apoptosis regulating glutamate excitotoxicity. Studies have also suggested that transient activation of syn-NMDAR and eNMDAR is a neuronal protective phenomenon without excitotoxic effects [58].

mGluRs and KARs are two other glutamate receptors. The mGluRs belong to the metabolic ionic receptor, which is a class of G protein-coupled receptors widely distributed within the CNS, with eight subtypes. Based on amino acid sequence homology, pharmacological properties, and intracellular signal transduction characteristics, mGluRs are divided into three groups: Group 1 contains mGluR 1 and mGluR 5, Group 2 contains mGluR 2 and mGluR 3, and Group 3 contains mGluR 4, mGluR 6, mGluR 7, and mGluR 8 [59]. Group 1 is postsynaptic receptors, which participate in excitotoxicity and the regulation of synaptic plasticity by activating a variety of downstream signaling pathways [60]. In addition, the physiological characteristic of Group 1 is a neuroexcitoenhancing effect, which can increase Ca^2+^ influx. Group 2 receptors participate in the regulatory process of synaptic plasticity by inhibiting voltage-gated calcium channels to reduce neuronal excitability. Group 3 is autoregulated presynaptic receptors that control the collection and release of synaptic glutamate [51].

KAR is a third class of ionotropic glutamate receptors, but no study has demonstrated a direct link between KAR and language function. KAR binding to kainic acid (KA) generates neuronal membrane depolarization leading to Ca^2+^ influx and triggering excitotoxic neuronal death cascade events [61]. KAR may play a role in maintaining different aspects of semantic memory, and the regulation of mGluRs may impact PSA.

**Figure 2:**
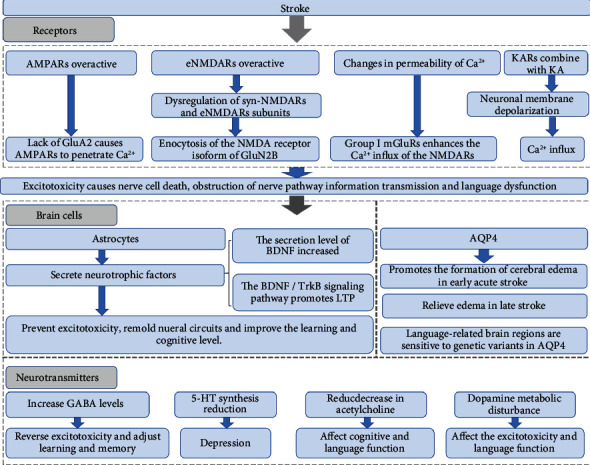
Brain cell, receptor, and neurotransmitter responses after a stroke. AMPAR: *α*-amino-3-hydroxy-5-methyl-4-isoxazole-propionic acid receptor; NMDA: *N*-methyl-D-aspartate; Ca^2+^: calcium ion; mGluR: metabotropic glutamate receptor; eNMDAR: extrasynaptic NMDAR; KA: kainic acid; BDNF: brain-derived neurotrophic factor; TrkB: tyrosine kinase receptor B; LTP: long-term potentiation; AQP4: aquaporin 4; GABA: *γ*-aminobutyric acid; 5-HT: 5-hydroxytryptamine.

In short, glutamate receptors may modulate language function in patients after stroke by affecting synaptic signaling and excitotoxicity (Figure 2).

### 2.4. Brain Cells

Glial cells, particularly astrocytes, can help maintain cellular environmental homeostasis and prevent excitotoxicity (Figure 2). Astrocytes are involved in various neuronal excitability and stroke pathogenesis processes through their interactions with neurons [62]. As supporting cells in the CNS, astrocytes have a large number of carrier proteins or ion channels related to glutamate uptake and transport on their cell membrane, which plays a vital role in the dynamic balance of the synthesis, decomposition, uptake, and release of glutamate in the brain [53].

The functional compensation of the healthy brain to the affected brain after stroke, i.e., the functional recovery caused by the remodeling of neural circuits by neural plasticity, is one of the mechanisms of language function recovery in PSA patients. There is a tight structural and functional link between astrocytes and neurons. Astrocytes can secrete multiple neurotransmitters involved in synapse sprouting and promote synaptic connectivity and regeneration. As glial cells are essential for maintaining the stable activity of brain neurons, astrocytes also play an essential role in neuronal remodeling and functional recovery after stroke. Neurotrophins are essential in synaptic remodeling, promoting axon regeneration, and repairing nerve damage. In ischemia and other stress states, astrocyte activation releases various neurotrophic factors (NTFs) to regulate synaptic formation and excitatory synaptic transmission, and mediating functional recovery.

Brain-derived neurotrophic factor (BDNF) increases its secretion levels after stroke onset and protects the nerves by reducing the excitotoxic effects of glutamate. As one of the BDNF-specific receptors of tyrosine kinase receptor B (TrkB), BDNF promotes LTP through TrkB signaling channels and participates in learning and memory. BDNF binding to TrkB maintains multiple classes of neuronal survival by activating downstream pathways promoting axonal growth and promoting synaptic plasticity to dendrites and axon growth.

The Val66Met single nucleotide (rs6265) is a guanine–adenine (G–A) base substitution in the coding region of the BDNF gene and has a polymorphic site for methionine (Met) [63]. Polymorphisms in the BDNF gene are thought to be associated with the prognosis of PSA. The atypical BDNF genotype (Met allele carriers) is more severe than the typical BDNF genotype (Val/Val) after aphasia [64]. However, some studies showed that carriers of the Met allele did not show significant differences in language recovery early after a stroke compared with noncarriers [65]. However, a later study stated that this result might be due to the activity-dependent BDNF secreted by the BDNF genotype taking a long time to act and be detected [66].

Aquaporin 4 (AQP4) is a water transporter protein that connects water to astrocytes and is the main water channel in the brain. It is mainly expressed in astrocytes and is associated with neuroplasticity in the brain. The bidirectional water transport by AQP4 promotes the formation of cytotoxic cerebral edema in the early stage of an acute stroke, leading to astrocyte swelling and hydrocephalus, causing neuroinflammation and cell death. In contrast, AQP4 promotes the elimination of angiogenic cerebral edema in the late stage of stroke [67]. Inhibiting AQP4 can reduce edema in brain tissue, and thus achieve relief from acute cerebral ischemic injury [68]. Furthermore, brain plasticity is associated with AQP4 and with language function. Language-related brain regions are sensitive to genetic variation in AQP4, where the single-nucleotide polymorphism site rs162008 of the AQP4 gene is associated with cortical GM volume changes. In individuals who learn a second language, gray matter volume (GMV) increases in the left posterior inferior frontal cortex and fusiform cortex [69].

Collectively, astrocytes play an essential role in maintaining brain function, limiting lesion expansion through anti-excitotoxicity effects, and releasing neurotrophins to promote synaptic reconstruction and nerve conduction pathway remodeling. Furthermore, astrocyte-specific water channels regulate cerebral water balance, thus providing benefits for neuroprotection.

### 2.5. Neurotransmitters

After a stroke, the balance of various neurotransmitters is disrupted (Figure 2). The regulation of various neurotransmitters can intervene in PSA recovery by promoting brain plasticity and LTP. Modern pharmacological modulation of the neurotransmitter system also affects PSA recovery to varying degrees. In addition to glutamate, other neurotransmitters are also important factors affecting the prognosis of PSA.

*γ*-Aminobutyric acid (GABA) is an important inhibitory neurotransmitter in the CNS. Extracellular GABA content was significantly increased during cerebral ischemia. By binding to specific transmembrane receptors in neurons (pre- and postsynaptic), GABA plays an inhibitory synaptic regulatory role in the brain's CNS and reverses neuronal damage caused by excitotoxicity [70]. Changes in GABA content and receptor function in the brain are crucial for many factors in learning and memory. GABA regulates learning and memory mainly in two ways. On the one hand, when the GABA content in the brain is reduced or receptor dysfunction occurs, proper supplementation and repair of GABA function can improve cognitive deficits. On the other hand, GABA causes abnormal activity and functional defects at the neural network level when excitotoxic injury occurs. When GABA is activated, it suppresses the neurotoxic effects caused by abnormal glutamate excitation and improves the decline in learning and memory function caused by neural abnormalities [71].

5-Hydroxytryptamine (5-HT) and norepinephrine (NE), another class of central neurotransmitters, are related to the treatment of depression. Cerebral apoplexy impairs the frontotemporal–basal ganglia–ventral brain stem loop, impairs the 5-HT and NE transmitter pathways, and reduces 5-HT and NE synthesis, leading to depression [72]. Serotonin plays an important role in regulating plasticity. Serotonin can promote LTP by enhancing NMDAR activation. In addition, activation of 5-HT receptors stimulates interneurons and astrocytes [73].

Central system acetylcholine transmitters are essential for cognitive functions, such as attention, memory, language, and thought judgment. The relevant transmitter pathway is impaired after a stroke, reducing acetylcholine transmitter levels in specific brain functional areas, and causing cognitive impairment and different degrees of impairment of language function [74]. Donepezil, acting as a reversible acetylcholinesterase inhibitor, improves language function by increasing acetylcholine concentrations in the cerebral cortex and basal ganglia nerve synapses.

The regulatory mechanism of dopamine after a stroke is complex and controversial. On the one hand, ischemia and reperfusion can lead to the metabolic disturbance of dopamine in the brain and affect excitotoxicity [75]. On the other hand, dopamine is commonly used to treat motor inability syndrome in patients with Parkinson's disease. Later, with the regulation of dopamine in speech and vocalization, dopamine was gradually applied to treat aphasia [76]. Dopamine is mainly distributed in the black substance, corpora interpeduncular, and hypothalamus. It is widely believed that the mesolimbic and mesocortical dopamine systems play a role in learning and memory. The mechanism which dopamine regulates PSA recovery has not been yet clearly defined. Dopamine may promote the activation and initiation of language output by stimulating the ascending midbrain–cortical pathway and enhancing output activity in impaired brain areas in aphasia [77]. Common dopamine drugs used to treat PSA are levodopa, the dopamine agonist bromocriptine, and amphetamine. However, Gill and Leff [78] analyzed 15 studies about dopaminergic therapy for PSA and proposed that the evidence for the efficacy of dopaminergic therapy on aphasia is not clear. Nevertheless, this may be due to the limited data for the drugs mentioned above for PSA [79]. Although dopamine regulates the vocal production circuits, it does not currently have a clear advantage in the treatment of aphasia.

## 3. Acupuncture in PSA

**Figure 3:**
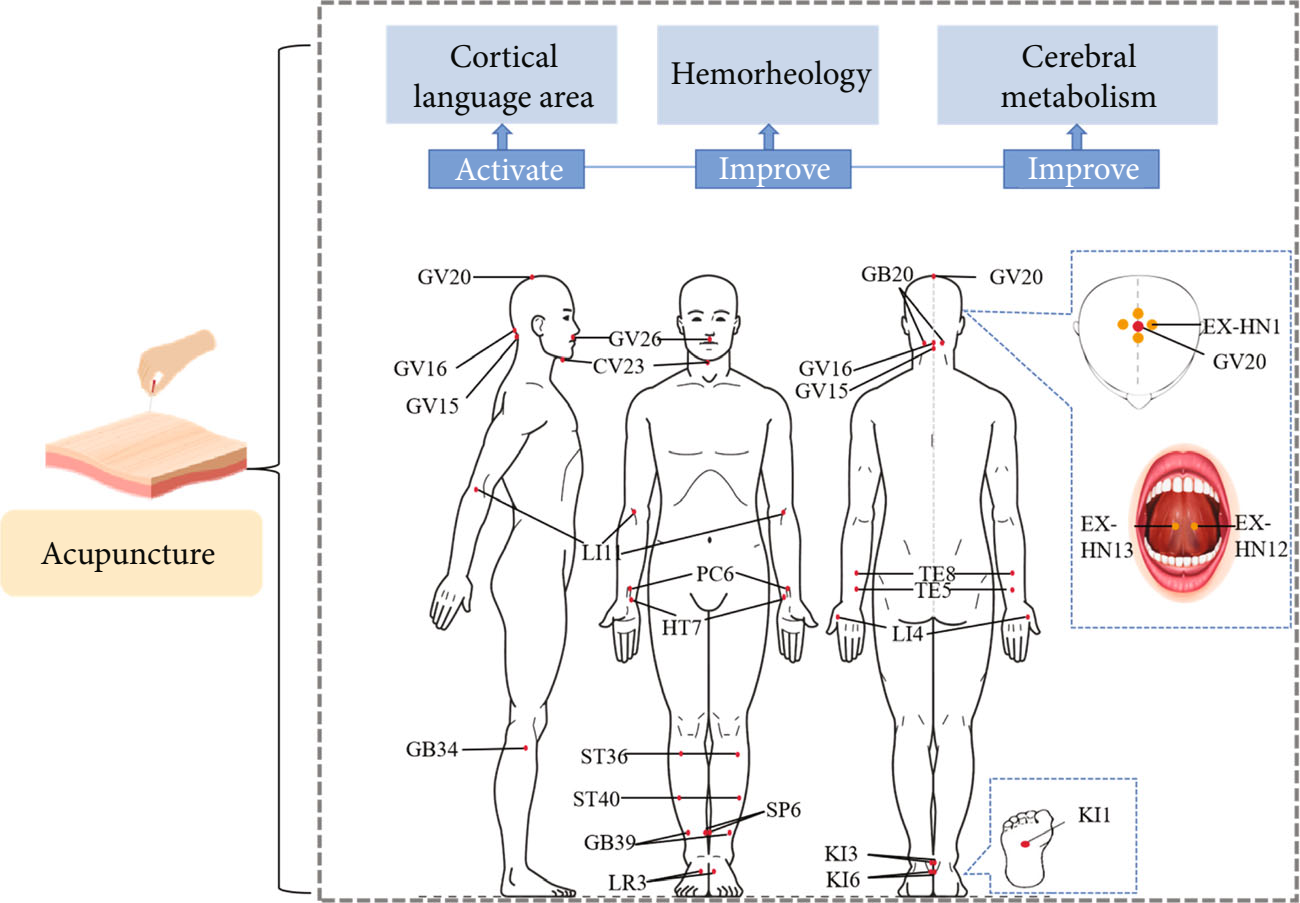
Effect of acupuncture in PSA patients. When acupuncture acts on acupoints, it will affect the recovery of aphasia from the cortical language area, hemodynamics, and brain metabolism. GV20: Baihui; EX-HN1: Sishencong; GV26: Shuigou; GV15: Yamen; GV16: Fengfu; GB20: Fengchi; EX-HN12: Jinjin; Yuye: EX-HN13; CV23: Lianquan; SP6: Sanyinjiao; ST40: Fenglong; ST36: Zusanli; LR3: Taichong; KI3: Taixi; GB34: Yanglinquan; GB39: Xuanzhong; KI6: Zhaohai; KI1: Yongquan; TE5: Waiguan; PC6: Neiguan; LI4: Hegu; LI11: Quchi; TE8: Sanyangluo; HT7: Shenmen; PC7: Daling; HT5: Tongli.

The recovery mechanism of PSA is widely related to various factors, such as compensation of the healthy brain, neuroplasticity, edema elimination of peripheral brain tissue, cerebral revascularization, and rapid reorganization of brain structure and function [80]. Acupuncture can effectively improve the functional communication of PSA, and thus improve the prognosis of it. The current study on acupuncture in PSA mainly focuses on activating cortical regions, blood rheology, and brain metabolism (Figure 3).

In addition, there is a time-limited window of neuroplasticity opened following stroke. Acupuncture treatment could enhance the role of neuroplasticity during this period, in which may be the key to acupuncture enhancement of neuroplasticity for improving PSA. However, the recovery mechanism of acupuncture in aphasia is still unclear. The mechanism where acupuncture affects neuroplasticity due to treatment of aphasia remains unclear. Synaptic plasticity and GM and WM plasticity may be one of the ways that acupuncture improves language function in PSA patients.

### 3.1. Activation of Cortical Language Areas

The therapeutic effect of acupuncture is closely related to acupoints. Acupoints are the principal place to transmit acupuncture stimulation and can cause the activation of different brain areas [81]. Different acupuncture techniques and whether acupuncture elicits the “Deqi” effect on the brain regions will be different [82]. Studies have shown that nerves, sensors, and musculoskeletal and vascular tissue are essential parts of acupoints [83]. Both the immediate and distant effects of acupoints are helpful for the recovery of aphasia [84], and both central and peripheral mechanisms may contribute to the therapeutic role of acupuncture [85]. For PSA patients, acupoints effectively stimulate language areas in the cerebral cortex [86]. Numerous studies have confirmed that the role of acupuncture is to produce specific changes between acupoints and brain activation regions. These changes can be demonstrated with the help of neuroimaging [87–89]. Acupuncture stimulation supports the modulatory effect of acupoints on neural matrix activity of the brain and improves language function by activating the language function mirror regions of specific language regions (homologous language regions in the right brain hemisphere) [90] (Table 1).

A previous study [91] found that acupuncture stimulation of Sanyangluo (TE8) can activate the right insula, the left precentral gyrus, and the right median cingulate and paracingulate gyri of the limbic lobe, suggesting that acupuncture has therapeutic effects in patients with PSA. This study is the first to demonstrate that when acupuncturing language-implicated acupoints, such as TE8, language-implicated brain regions in PSA patients can be selectively activated, providing evidence for exploring acupuncture in PSA recovery.

Shenmen (HT7) and Daling (PC7) activate brain regions that are not identical, but both activate language-implicated brain regions [92]. The coactivated Brodmann areas were Brodmann areas 22, 40, 44, and 47. Brodmann area 22 is one of the classical Wernicke's areas involving language understanding. Brodmann area 40 is associated with semantic processing and linguistic repetition. Brodmann area 44 involves motor language and refers to the grammatical system. Brodmann area 47 is one of the critical components of the language system, coordinating with other subcortical language centers involved in language formation and semantic processing [93–99].

In addition, Tongli (HT5) and Xuanzhong (GB39) were also used to treat PSA. EA at HT5 and GB39 influences language cognitive functions by mobilization of the frontal lobe, temporal lobe, parietal lobe, and limbic system [100]. According to the acupuncture theory, the left brain will be activated when acupuncturing on the right side of the body. Multiple language-related brain regions could be activated when stimulating the right HT5, such as the left insula, right middle frontal gyrus, and bilateral central temporal gyrus [101]. Therefore, acupuncturing the right HT5 acupoint results in activation of the bilateral language-related areas. In addition, hormone levels were increased accordingly. Furthermore, another study also induced the activation of the relevant language network regions by performing electrical stimulation of HT5, affirming the therapeutic effect of HT5 for PSA [102].

### 3.2. Hemorheology

In the last century, researchers have found that aphasia recovery is closely associated with cortical hypoperfusion [104]. Abnormal hemorheology [blood viscosity, plasma viscosity (PV), and hematocrit], a significant risk factor for stroke, can cause a microcirculation disorder and thrombosis. Aphasia is an early sign of adverse results in patients with mild ischemic stroke. Increased blood operation resistance, slow blood flow speed, and elevated blood viscosity and PV are essential pathological bases for the formation of aphasia after a stroke [105–108]. Furthermore, elevated PV leads to a significantly increased risk of occlusive cerebrovascular disease [109]. The brain is a complete network, and changes in local vascular supply may disrupt the integrity of the entire region. Although it is unclear how changes in hemorheology respond to potential neuronal activity, monitoring changes in hemorheology provides a new direction for PSA recovery [110].

Acupuncture therapy can reduce whole blood viscosity to increase the elasticity of cerebral arteries, reduce vascular tension, and improve blood viscosity and aggregation to improve cerebral blood flow and promote blood circulation [111]. Therefore, acupuncture can promote the recovery of language function in patients with PSA by affecting hemorheology. This result shows that acupuncture therapy has a particular impact on hemorheology in the brain, which may improve cerebral blood flow, promote brain tissue metabolism, nourish the nervous system, and thus repair the local nerve function of the body. Recent studies [112] have used acupuncture combination treatment to treat patients with PSA, and hemodynamic parameters improve after treatment; the results further affirm the previous trial results.

### 3.3. Improvement of Cerebral Metabolism

Brain activity is closely related to cerebral blood flow and cerebral oxygen metabolism rate. Reduced cerebrovascular reactivity is associated with an increased risk of cerebral ischemic disease [113]. The change in cerebral blood flow velocity is a reliable indicator reflecting the altered perfusion area of the large cerebral artery and one of the indicators to predict language function in patients with PSA [114–116]. Hypoperfusion in subcortical language function areas will directly affect language function in PSA patients [117]. Some studies summarized the frequently used acupoints and how they ameliorate PSA. Among these acupoints, Lianquan (CV23), Baihui (GV20), Yamen (GV15), Jinjin, and Yuye (EX-HN12 and EX-HN13) are the most essential and frequently used acupoints in PSA [118] (Table 1).

The scalp needle is based on the functional positioning of the cerebral cortex and divides the corresponding stimulation area on the scalp for acupuncture. The scalp needle acts on the head and has some role in stimulating related nerve tissue and activating nerve reflexes in different brain regions [119, 120]. Earlier studies suggested that scalp needles can improve brain oxygen supply by accelerating the recovery of cortical function, and thus increasing blood perfusion in the damaged cerebral hemispheres [121]. The tongue is an integral part of language tasks and is rich in blood vessels, lymph, and neural networks. A domestic study that examined hemodynamic changes in 95 stroke patients found that tongue needles can help improve blood circulation, restore hemodynamic balance, and prevent thrombosis [122]. Therefore, tongue needle treatment can promote the recovery of language function by improving blood flow in cerebral ischemic areas. Body acupuncture emphasizes holistic treatment. Body acupuncture can combine syndrome differentiation with meridian differentiation and regulate the mind and Qi [123, 124].

In most cases, acupuncture achieves better results through the synergistic effect of acupoint combination. Acupuncture in PSA attaches great importance to the location of the acupoint selection, mostly adopting local point selection, mainly head and neck acupoints, combined with the trunk and limb acupoints to achieve a better effect.

### 3.4. Depending on Neuroplasticity to Improve Functional Recovery

#### 3.4.1. Synaptic Plasticity

*(1) AMPA Receptors*. Loss of the GluA2 subunit in AMPARs after stroke leads to excitotoxicity. This effect affects the transmission of information in neurons and finally causes dysfunction after stroke. However, electroacupuncture (EA) pretreatment can upregulate GluA2 expression by activating the endocannabinoid system [125]. The cannabinoid CB1 receptor increases GluA 2 expression levels to inhibit the permeability of the postsynaptic membrane to Ca^2+^ and ultimately weakens excitotoxicity to protect neurons. EA pretreatment reduced the infarct volume, inhibited neuronal apoptosis after reperfusion, and improved neurological outcomes [126].

*(2) NMDA Receptors*. In a previous case report, a stroke patient treated with NMDA receptor antagonists for pseudobulbar affect was unexpectedly found to have significantly improved speech function and largely restored social ability. These findings suggest that inhibiting the excitotoxic effects of neurotransmitters and improving neural pathway signaling in the brain are of great significance for the recovery of speech function in patients with aphasia [127].

Even if the cell death signaling pathway is activated under excitotoxicity, enhancing the channel function of NMDARs containing the GluN2A subunit can still promote the enhancement of survival signaling, and thus enhance the survival rate of neuronal cells [128]. The NMDA receptor regulation of the activated survival pathway or the death pathway depends on the balance of the syn-NMDAR and eNMDAR subunits. This may affect the transmission of subcortical language information. This finding has significant implications for subsequent studies of excitotoxicity. Hippocampal LTP expression in stroke patients is suppressed, and learning, memory, and language functions are impaired. Existing studies have shown that acupuncture treatment reduces excessive neuronal excitatory amino acids (EAAs) in brain tissue. EA therapy found that acupuncture reduces the activity of protein kinase A (PKA) by activating the 5-hydroxytryptamine receptor 1A, leading to phosphorylation of AMPA receptor and NMDA receptor, thus activating syn-NMDAR and promoting LTP [129]. The cAMP/PKA signaling pathway also regulates NMDAR-dependent LTP [130]. Meanwhile, animal experiments also found evidence of improved learning and memory after cerebral infarction by acupuncture through the cAMP/PKA/CREB signaling pathway [131]. Acupuncture antagonizes excitotoxicity damage by activating syn-NMDAR, and enhancing neuronal survival signals this way may be advantageous over conventional NMDA receptor antagonists. Moreover, acupuncture for cerebrovascular disease involves reducing the excitotoxic effects by enhancing syn-NMDAR and inhibiting eNMDAR, thus reducing the intracellular Ca^2+^ concentration [132]. Acupuncture treatment can antagonize hippocampal nerve cell apoptosis, reduce the degree of damage to secondary nerve cells, and promote the repair of damaged neurons, thus reducing ischemia and reperfusion injury, protecting brain tissue, and improving brain function [133].

*(3) Metabolotropic Glutamate Receptors*. mGluRs are mainly involved in excitotoxicity and regulate synaptic plasticity. Among several subtypes of mGluRs, the neuroprotective effect observed in patients with neurodegenerative diseases was mainly dependent on one group of mGluRs. Activation by its selective agonist (S)-3,5-dihydroxyphenylglycine leads to a reduced susceptibility of nerve cells to NMDA-mediated damage [134]. Activation of mGluR5 can reduce apoptosis in ischemic tissue and plays a protective role. Acupuncture can not only elevate mGluR5 expression, but also reduce mGluR1 mRNA expression to reduce the receptor number [135]. Its sensitivity exerts a neuroprotective effect on ischemic brain tissue and finally alleviates the neurotoxicity of excitatory amino acids [136].

In summary, it can be reasonably speculated that acupuncture may be a pathway that regulates the expression and activity of glutamate and its receptors and reduces excitotoxicity to interfere with cell death in the penumbra region to improve the survival rate of penumbra cells and protect neurons. Furthermore, it can reduce the imbalance of glutamate neurotransmitter language information transmission to protect the integrity of the language signal transduction pathway and reduce the volume of the infarction site. In addition to reducing the infarct volume and improving the prognosis of stroke patients, acupuncture/EA preconditioning is of great significance in preventing secondary infarction and improving the prognosis of stroke patients. Thus, acupuncture is a means of treatment and reflects the idea of curing the disease.

*(4) Neurotrophins*. Glutamate and astrocytes are essential for maintaining neuroplasticity in the brain [137]. Glial glutamate transporter-1 (GLT-1) is the primary transporter for glutamate uptake by astrocytes. It is responsible for removing the primary transporter of excess glutamate from the extracellular space, thereby reducing the excitotoxicity of glutamate to the CNS and exerting a neuroprotective role [138]. Animal trials have shown that EA can protect the nerve by upregulating GLT-1 expression and inhibiting excessive glutamate uptake to produce ischemic tolerance [139]. In addition, astrocytes can uptake extracellular glutamate through the excitatory amino acid transporters 1 (EAAT1) and 2 (EAAT2) on the cell membrane surface, transforming it through the action of glutamine synthetase (GS) to produce glutamine, thus reducing the concentration of glutamate in the synaptic gap, and thereby reducing the excitotoxic effect [140].

BANF is activated and released by astrocytes during poststroke stress. BANF drives neuroplasticity, promoting cortical language regions regulating PSA recovery [66]. Improving BDNF levels in peripheral serum can promote language learning ability and cognitive function [141]. In addition, acupuncture treatment has been demonstrated to promote the reconstruction of neural circuits and synaptic plasticity in treating neurodegenerative lesions [142]. In addition, EA treatment can promote neuronal survival and can positively act on synaptic plasticity by activating the conduction of the BDNF/TrkB signaling pathway [143]. Therefore, we can speculate that acupuncture treatment can promote the expression of BDNF protein in the cerebral ischemic region of stroke patients, thus promoting the survival of brain cells and the reconstruction of neuronal circuits to increase the number of dendritic spines in hippocampal neurons, promoting synaptic remodeling, and enhance synaptic plasticity to promote the recovery of language function.

*(5) γ-Aminobutyric Acid*. Individuals with higher concentrations of GABA have better semantic processing power [144]. Modern pharmacology indicates that GABA has mixed effects on aphasia, including delayed aphasia recovery and improved language function [145]. Acupuncture can be neuroprotective by inhibiting the excitotoxic effects of glutamate by activating the expression activity of GABA [146, 147]. Moreover, the underlying mechanism of the neuroprotective effect of GABA may be related to the activation of the GABAB receptor-cAMP/PKA/CREB signal transduction pathway [148]. Therefore, acupuncture improvement of language function in PSA patients may be due to the upregulation of GABA expression by acupuncture treatment, activating the cAMP/PKA/CREB signaling pathway, and inhibiting excitotoxic effects. However, other studies show that GABA plays a negative role in regulating learning and memory, and inhibiting the GABAB receptor can effectively improve the lack of function of patients and plays a neuroprotective role [149].

*(6) 5-Hydroxytryptamine*. The role of 5-HT for aphasia emerged in earlier studies [150]. The idea that antidepressants can be used to treat aphasia has been postulated before [145]. Although minor studies discuss the language function reconstruction in PSA by 5-HT, the promoting effect of 5-HT on the expression of BDNF may be an essential breakthrough. The efficacy of acupuncture in treating depression through regulating 5-HT levels has been demonstrated [151, 152]. Therefore, it also provides us with new ideas to explore the neural mechanism of acupuncture for PSA.

*(7) Acetylcholine*. Acetylcholine is required for LTP induction in vivo. Cholinergic potentiation therapy can facilitate the recovery of language function in PSA patients by improving structural plasticity in WM [153]. Acupuncture can impact learning and memory by enhancing acetylcholine levels in the brain, and it may have similar regulatory principles on the prognosis of PSA [154].

**Figure 4:**
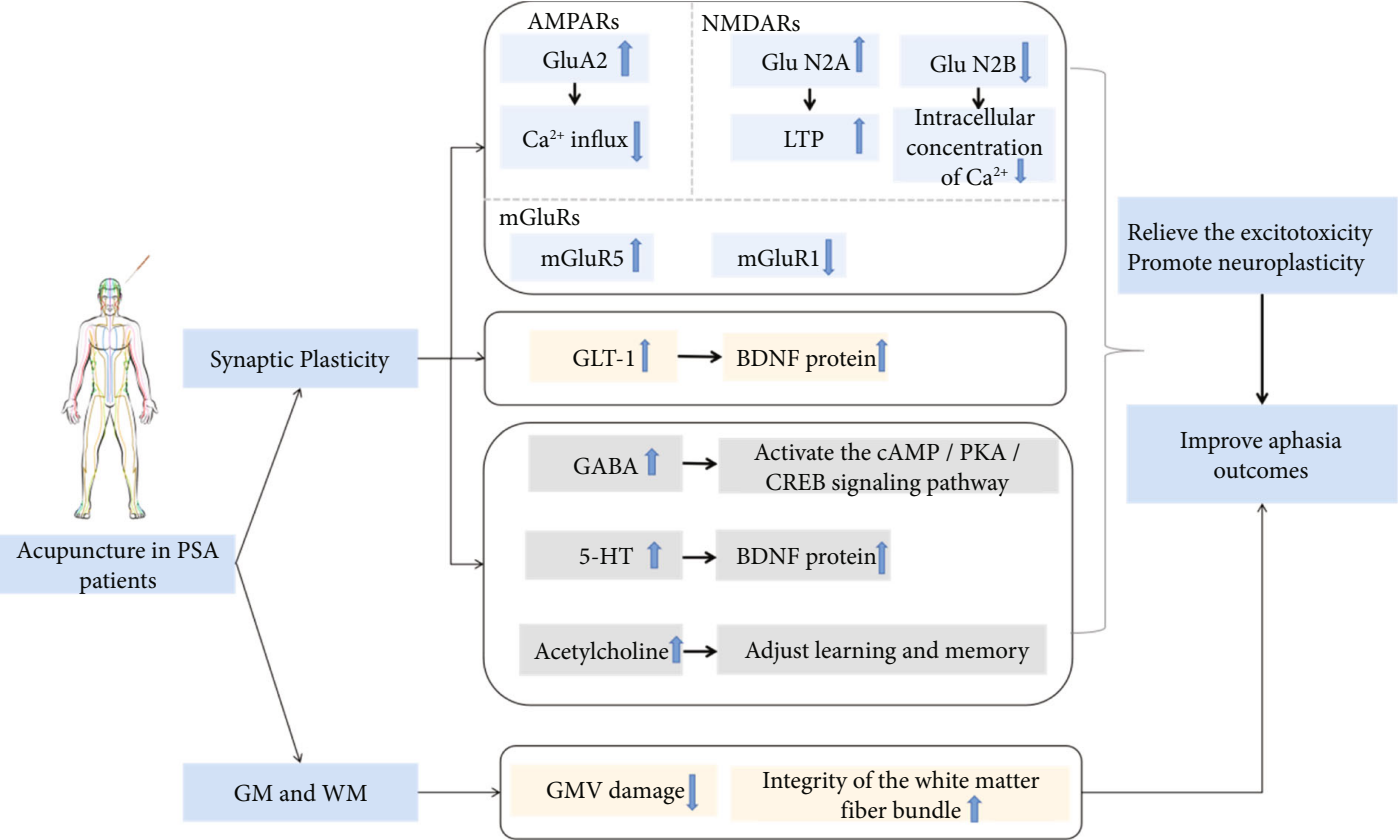
The possible positive effect of acupuncture on PSA. AMPAR: *α*-amino-3-hydroxy-5-methyl-4-isoxazole-propionic acid receptor; NMDAR: *N*-methyl-D-aspartate receptor; Ca^2+^: calcium ion; mGluR: metabotropic glutamate receptor; GLT-1: glial glutamate transporter-1; BDNF: brain-derived neurotrophic factor; LTP: long-term potentiation; GABA: *γ*-aminobutyric acid; 5-HT: 5-hydroxytryptamine; GM: gray matter; WM: white matter; GMV: gray matter volume.

In conclusion, acupuncture plays an important role in synaptic plasticity by promoting synaptic activity and remodeling through regulating glutamate receptors, NTFs, and other neurotransmitters (Figure 4).

#### 3.4.2. GM and WM Integrity

Language processing requires an interconnected network. The integrity of the brain WM pathways concerns the prognosis of aphasia, and a combination of WM integrity or GM and WM integrity can better predict aphasia recovery [155]. Language formation depends on a specific brain area and brain interval interconnected WM fiber bundle and language processing depends on WM fiber information between different cortical areas. WM fibers connecting different subcortical areas involved in language function are different [156]. After a stroke, WM pathway integrity is destroyed, the WM fiber connection is interrupted, and disordered development of the formation and processing of language function leads to PSA development. Nevertheless, acupuncture can improve impaired language function by improving the microstructure of WM conduction bundles related to language and memory and enhancing cortical information communication [157].

The alteration of GM is strongly associated with language comprehension dysfunction in PSA patients. Cortical GM structure changes with intrinsic functional connectivity (IFC) after a stroke and changes in GM structure are associated with IFC changes [158]. The GM structural changes in aphasia include GMV atrophy in the corresponding language function area of the lesion side of the brain and a compensatory increase in GMV in the language backup area of the lesion side and the language mirror area of the healthy brain [159]. The intervention effect of acupuncture treatment on brain GM injury in stroke patients can effectively advance the progression of brain GM injury after stroke [160]. Furthermore, acupuncture treatment can promote the reorganization of brain structure in stroke patients, thus improving the quality of life of stroke patients [161]. After acupuncture treatment, the resting-state functional magnetic resonance scan showed significant changes in GMV. With significant increases in GMV in the left frontal and MTG regions, GM density also increased in multiple brain regions after acupuncture treatment [162].

Overall, the mechanism of language function recovery caused by acupuncture treatment may reduce the volume of GM damage and promote the repair of the structural integrity of the WM fiber bundle (Figure 4).

## 4. Discussion

Acupuncture has become an important therapeutic tool for PSA, and its improvement of poststroke language function has shown its unique advantages clinically. Acupuncture treatment techniques and acupuncture points are diverse, and the addition of acupuncture manipulation quantity theory is also a new tool to further enhance the effectiveness of acupuncture treatment. Language is an essential ability in our daily life. PSA is one of the most common cognitive impairments after a stroke [163]. Thus, the related functions of language usually have an essential role in our daily psychological activities and cognitive processes.

We reviewed previous research and found that, based on two models of aphasia, the research on the mechanism of acupuncture treatment in PSA mainly concerns: (1) the single acupoint for the activation of different cortical areas in aphasia patients; and (2) acupuncture improves blood circulation, increases cerebral blood flow, restores the blood supply of damaged brain tissue, and improves hemorheology. Although the efficacy of acupuncture for PSA is significant and has been widely used in clinical practice, the exact recovery mechanism of acupuncture for PSA improvement is unclear.

Neuroplasticity plays an irreplaceable role in the recovery process of PSA. Neuroplasticity recovery for promoting aphasia involves the recovery of recruiting residuals and reorganizing new neural mechanisms, such as establishing alternative functional networks, synaptic remodeling, and axonal sprouting.

**Figure 5:**
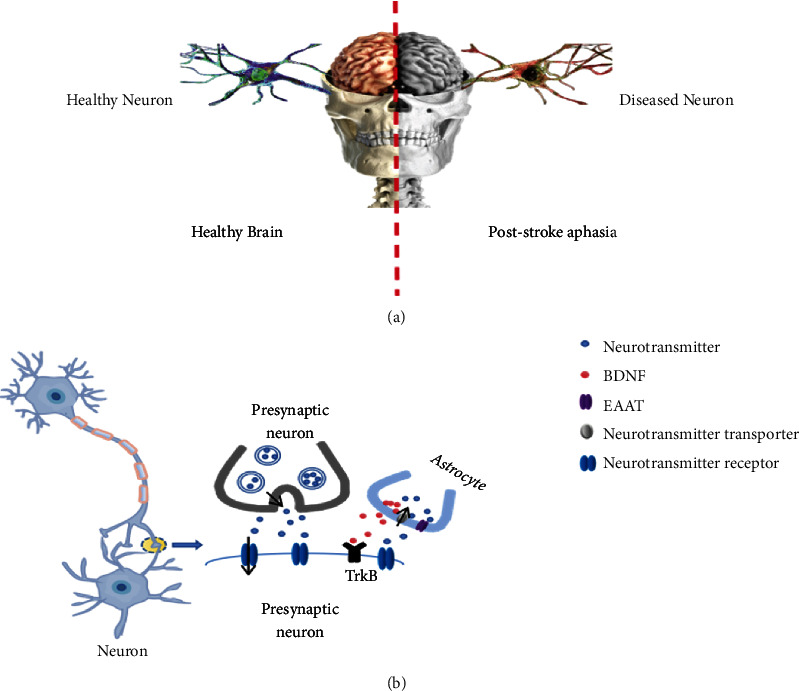
The neuronal changes after PSA. (a) Compared with the healthy brain, the regular neuronal activity in PSA patients was blocked and replaced by pathological neurons. (b) In the healthy brain, neurotransmitters signal between synapses; BDNF secreted by astrocytes binds to TrkB to participate in synaptic growth, and EAAT maintains extracellular glutamate homeostasis. Neurotransmitter conduction is blocked, and the availability of neurotransmitters on synapses is reduced after stroke. The balance of neurotransmitter activity is broken, impairing the integrity of the neural pathway. Thus, dysfunction occurs.

The recovery of function is the basis of functional language acquisition. The integrity of the GM and WM pathways is essential for the prognosis of brain language function. Furthermore, neurotransmitters have a critical role in working memory function. Based on this, we analyzed the underlying mechanisms of PSA recovery and the infinite possibility of acupuncture affecting this recovery from the perspective of synaptic plasticity as well as GM and WM integrity. After a stroke, the excessive release of glutamate causes excitotoxicity to have an inhibitory effect on the body and blocks the normal conduction of the neural pathway, reduces neurotransmitter conduction activity in the brain, and disrupts the integrity of the pathway, which may lead to the occurrence of language dysfunction (Figure 5) [51]. NMDA is predominant among the numerous glutamate substances released and bidirectionally regulates neurons. The balance between GABA acting as an inhibitory neurotransmitter and excitatory glutamate is critical for functional maintenance. Acetylcholine, however, regulates synaptic plasticity by promoting NMDA delivery to both glutamatergic and dopaminergic cells to promote LTP [164].

Evidence has demonstrated the exact efficacy of acupuncture therapy on CNS plasticity. Acupuncture-induced neuroplasticity is associated with neurotransmitters and NTFs, which may be the potential molecular basis for promoting aphasia recovery. Neuroplasticity is an important bridge to promote the recovery of neurodegenerative lesions [165]. Almost all treatments for aphasia involve learning and memory [166]. Acupuncture for PSA may involve multiple brain mechanisms related to cognitive processes, including determining stimulus salience, regulating cognitive control, restoring language networks, improving aphasia outcomes, promoting neuroplasticity, alleviating brain edema and tissue metabolic disorders, and increasing synaptogenesis in compensatory language circuits [167].

## 5. Conclusion

Although the increasing number of neuroimaging studies on PSA have significantly advanced the knowledge on the mechanisms of PSA recovery, many issues remain unresolved regarding the mechanism of acupuncture on PSA. We analyze the literature and propose that the language recovery mechanism under acupuncture may involve two points: (1) glutamate receptors and neurotransmission of neurotransmitters may play a key role in the normal physiological process of language and the transmission of language information; (2) astrocytes exert an effect on neural plasticity through cellular homeostasis, and therefore intervene in the recovery of language function. This cellular homeostasis improves the outcome of PSA by promoting synaptic reconstruction and remodeling nerve conduction pathway. This study emphasizes the effect of acupuncture, and how can glutamate receptors, neurotransmitters, and astrocytes shed light on the biological mechanisms of acupuncture and neuroplasticity, and increase our understanding how acupuncture affects specific language disorders by regulating dysregulation of neurotransmitters/astrocytes.

## Figures and Tables

**Table 1:** Acupoints possibly used in PSA.

Acupoint	Location [103]	Function
Head		
Baihui (GV20)	On the head, 5 B-cun superior to the anterior hairline on the anterior median line	Improve the oxygen supply and increase blood perfusion of the brain; Activate the language centers; and Regulate the mind and restore consciousness to speed up the repair of the language function area of the cerebral cortex
Sishencong (EX-HN1)	Four acupoints on the vertex of the head located 1 B-cun posterior, anterior, and lateral to GV 20	
Shuigou (GV26)	In the face, at the junction of the upper 1/3 and middle 1/3 of the philtrum groove	
Yamen (GV15)	In the posterior region of the neck, in the depression superior to the spinous process of the second cervical vertebra (C2), on the posterior median line	
Fengfu (GV16)	In the posterior region of the neck, directly inferior to the external occipital protuberance, in the depression between the trapezius muscles	
Fengchi (GB20)	In the anterior region of the neck, inferior to the occipital bone, in the depression between the origins of sternocleidomastoid and the trapezius muscles	

Tongue		
Jinjin and Yuye (EX-HN12; EX-HN13)	In the mouth, EX-HN12 is located with tongue furled, on the vein on the left side of the frenulum of the tongue. EX-HN13 is located on the vein on the right side of the frenulum of the tongue	Dredge the meridians and regulate Qi and blood
Lianquan (CV23)	In the anterior midline, above Adam's apple, the depression at the upper edge of the hyoid	

Lower limbs		
Sanyinjiao (SP6)	On the tibial aspect of the leg, posterior to the medial border of the tibia, 3 B-cun superior to the prominence of the medial malleolus	Combining syndrome differentiation with meridian differentiation and regulating the mind and Qi; Activate multiple language-related brain regions; and GB39 activates multiple language-related brain regions.
Fenglong (ST40)	On the anterolateral aspect of the leg, lateral border of the tibialis anterior muscle, 8 B-cun superior to the prominence of the lateral malleolus	
Zusanli (ST36)	Near the knee joint of the hind limb 2 mm lateral to the anterior tubercle of the tibia in rodents	
Taichong (LR3)	On the dorsum of the foot, between the first and second metatarsal bones, in the depression distal to the junction of the bases of the two bones, over the dorsalis pedis artery	
Taixi (KI3)	On the posteromedial aspect of the ankle, in the depression between the prominence of the medial malleolus and the calcaneal tendon	
Yanglinquan (GB34)	On the fibular aspect of the leg, in the depression anterior and distal to the head of the fibula	
Xuanzhong (GB39)	On the fibular aspect of the leg, anterior to the fibula, 3 B-cun proximal to the prominence of the lateral malleolus	
Zhaohai (KI6)	On the medial aspect of the foot, 1 B-cun inferior to the prominence of the medial malleolus, in the depression inferior to the medial malleolus	
Yongquan (KI1)	On the sole of the foot, in the deepest depression of the sole when the toes are flexed	

Upper limbs		
Waiguan (TE5)	On the posterior aspect of the forearm, midpoint of the interosseous space between the radius and the ulna, 2 B-cun proximal to the dorsal wrist crease	Combining syndrome differentiation with meridian differentiation and regulating the mind and Qi; TE8 activates the language regions; HT7 and PC7 activate the Brodmann areas 22, 40, 44, and 47; and HT5 activates multiple language-related brain regions and increase the hormone levels.
Neiguan (PC6)	On the anterior aspect of the forearm, between the tendons of the palmaris longus and the flexor carpi radialis, 2 B-cun proximal to the palmar wrist crease	
Hegu (LI4)	On the dorsum of the hand, radial to the midpoint of the second metacarpal bone	
Quchi (LI11)	On the lateral aspect of the elbow, at the midpoint of the line connecting LU5 with the lateral epicondyle of the humerus	
Sanyangluo (TE8)	On the posterior aspect of the forearm, midpoint of the interosseous space between the radius and the ulna, 4 B-cun proximal to the dorsal wrist crease	
Shenmen (HT7)	In the depression radial to the proximal border of the pisiform bone on the palmar wrist crease	
Daling (PC7)	On the anterior aspect of the wrist, between the tendons of palmaris longus and the flexor carpi radialis, on the palmar wrist crease	
Tongli (HT5)	On the anteromedial aspect of the forearm, radial to the flexor carpi ulnaris tendon, 1 B-cun proximal to the palmar wrist crease	

## Data Availability

No available data are requested.
